# SNHG1 promotes MPP^+^-induced cytotoxicity by regulating PTEN/AKT/mTOR signaling pathway in SH-SY5Y cells via sponging miR-153-3p

**DOI:** 10.1186/s40659-019-0267-y

**Published:** 2020-01-06

**Authors:** Jun Zhao, Lijiao Geng, Yong Chen, Chunfang Wu

**Affiliations:** 1 Department of Neurology, Huaihe Hospital of Henan University, No. 357 Ximen Street, Kaifeng, 475000 China; 2 Department of Rehabilitation Medicine, Huaihe Hospital of Henan University, Kaifeng, 475000 China

**Keywords:** IncRNA, SNHG1, miR-153-3p, PTEN, MPP^+^, Parkinson’s disease

## Abstract

**Background:**

Long non-coding RNA small molecule RNA host gene 1 (SNHG1) was previously identified to be relevant with Parkinson’s disease (PD) pathogenesis. This work aims to further elucidate the regulatory networks of SNHG1 involved in PD.

**Methods:**

1-methyl-4-phenyl-1,2,3,6-tetrahydropyridine-hydrochloride (MPTP)-induced mice and 1-methyl-4-phenylpyridinium (MPP^+^)-treated SH-SY5Y cells were respectively constructed as the in vivo and in vitro PD models. Expression levels of SNHG1 and miR-153-3p were detected by qRT-PCR. Protein expression levels of phosphate and tension homology deleted on chromosome ten (PTEN) were measured by western blotting assay. Cell viability and apoptosis were determined by MTT and flow cytometry assays. The interactions among SNHG1, miR-153-3p and PTEN were identified by luciferase reporter assay, RNA immunoprecipitation, and/or RNA pull-down analysis.

**Results:**

Increased SNHG1 expression was found in midbrain of MPTP-induced PD mice and MPP^+^-treated SH-SY5Y cells. Overexpression of SNHG1 lowered viability and enhanced apoptosis in MPP^+^-treated SH-SY5Y cells. Moreover, SNHG1 acted as a molecular sponge to inhibit the expression of miR-153-3p. Furthermore, miR-153-3p-mediated suppression of MPP^+^-induced cytotoxicity was abated following SNHG1 up-regulation. Additionally, PTEN was identified as a direct target of miR-153-3p, and SNHG1 could serve as a competing endogenous RNA (ceRNA) of miR-153-3p to improve the expression of PTEN. Besides, enforced expression of PTEN displayed the similar functions as SNHG1 overexpression in regulating the viability and apoptosis of MPP^+^-treated SH-SY5Y cells. Finally, SNHG1 was found to activate PTEN/AKT/mTOR signaling pathway in SH-SY5Y cells by targeting miR-153-3p.

**Conclusion:**

SNHG1 aggravates MPP^+^-induced cellular toxicity in SH-SY5Y cells by regulating PTEN/AKT/mTOR signaling via sponging miR-153-3p, indicating the potential of SNHG1 as a promising therapeutic target for PD.

## Background

As the second-most common neurodegenerative disease following Alzheimer’s disease (AD), Parkinson’s disease (PD) is a progressive aged-related central nervous system (CNS) disorder [1]. Typical pathological characteristics of PD contain the relatively-selective loss of dopaminergic (DA) neurons in the substantia nigra pars compacta (SNpc) of the brain, the decrease of dopamine secretion in the striatum pathway, and the formation of intracellular inclusions-Lewy body (LB) in the cytoplasm of the remaining dopamine neurons [2]. Major clinical symptoms of PD patients involve the changes of both non-motor and motor functions, such as the dyskinesia, dystonia, gait disorder and postural abnormality [3]. Many investigations demonstrate that PD might be induced by complex interactions between external environment factors and genetic alterations [4]. Mitochondrial function, α-synuclein proteostasis, oxidative stress, calcium homeostasis, axonal transport and neuroinflammation have been reported to be associated with PD pathogenesis [5]. Though enormous efforts have been dedicated to illustrate the molecular mechanisms of PD etiology, there still lacks effective targeted drugs and therapeutic strategies for PD patients.

Long non-coding RNAs (LncRNAs) are a group of transcripts longer than 200 nucleotides with limited protein-coding ability. LncRNAs have been extensively investigated due to their essential roles in various human diseases by regulating different biological events and cellular functions [6]. In recent years, lncRNAs are highlighted to be involved in the normal functioning of CNS, including brain development, maturation, differentiation, neuronal cell specification, neurogenesis, and neurotransmission [7]. Also, several lncRNAs are identified as the vital regulatory molecules in the pathophysiology of neurodegenerative diseases, including PD [8]. For instance, lincRNA-p21 suppressed viability and enhanced apoptosis of SH-SY5Y cells induced by 1-methyl-4-phenylpyridinium (MPP^+^) via down-regulating miR-1277-5p and up-regulating α-synuclein protein [9]. HOTAIR facilitated PD progression through modulating miR-126-5p and RAB3IP in a ceRNA-dependent manner [10]. Small molecule RNA host gene 1 (SNHG1, Genebank accession ID: 23642), also named as linc00057, is a relatively novel lncRNA transcribed from UHG and located in 11q12.3 region of the chromosome with approximately 11 exons, hosting eight small nucleolar RNAs (snoRNAs) from its spliced introns. SNHG1 has been demonstrated to facilitate the occurrence and development of various malignant cancers [11, 12]. Particularly, several studies reveal the involvement of SNHG1 in brain functions and CNS disorders. For instance, SNHG1 functioned as an oncogene in glioma through increasing cell proliferation and invasion, and repressing apoptosis [13]. Also, SNHG1 contributed to glioma progression via competitively binding to miR-194 to modulate PHLDA1 expression [14]. Besides, neuroprotective effects of SNHG1 on survival and angiogenesis of brain microvasular endothelial cells (BMECs) in ischemic stroke are dependent on miR-199a [15], miR-338 [16] and miR-18a [17] through regulating HIF-1α. Interestingly, a previous research showed that SNHG1 expression was increased in early brain stem type PD and then maintained at high levels during the course of PD [18]. Moreover, Chen et al. [19] reported that SNHG1 induced α-synuclein aggregation and excitotoxicity in human neuroblastoma SH-SY5Y cells by regulating miR-15b-5p/SIAH1 signaling axis. Cao et al. [20] found that SNHG1 contributed to neuroinflammation via regulating the miR-7/NLRP3 pathway. However, the relevant molecular mechanisms of SNHG1 involved in PD aetiology still need to be further clarified.

In recent years, hypothesis of competing endogenous RNAs (ceRNAs) refers to RNA transcripts that harbor miRNA response elements (MREs) can sequester miRNAs from other targets sharing the same MREs, thereby regulating their expressions [21]. Here, we aim to elucidate the gene regulatory networks of SNHG1 as a ceRNA in PD pathogenesis.

In this study, we confirmed that SNHG1 expression was up-regulated in in vitro and in vivo models of PD. Moreover, SNHG1 overexpression aggravated neuronal injury in MPP^+-^treated SH-SY5Y cells by decreasing viability and promoting apoptosis. Furthermore, SNHG1 induced neurotoxicity in SH-SY5Y cells by stimulating PTEN/AKT/mTOR signaling via targeting miR-153-3p. Through in-depth exploration of the biological function and regulatory mechanism of SNHG1, it is expected to promisingly facilitate the clinical diagnosis and targeted therapy for PD patients.

## Methods

### PD animal model

A total of 20 healthy male C57BL/6 mice (10–12 weeks, 25–35 g) were purchased from Shanghai Laboratory Animal Center of Chinese Academy of Sciences (Shanghai, China). Mice were cultivated at constant temperature (22 ± 2 °C) and humidity under a 12 h light–dark cycle with free access to water and food. Mice were randomly assigned into 1-methyl-4-phenyl-1,2,3,6-tetrahydropyridine-hydrochloride (MPTP) group (n = 10) and control group (n = 10). For MPTP group, mice were intraperitoneally injected with 30 mg/kg MPTP hydrochloride (Sigma-Aldrich, St. Louis, MO, USA) for 7 consecutive days. For control group, mice received an equivalent volume of 0.9% sterile saline solution. At 7 days after the last injection, mice were killed with ventral midbrain containing SNpc isolated and conserved at − 80 °C for further analysis. Animal protocols in this study were performed in compliance with the Guide for Care and Use of Laboratory Animals of National Institutes of Health with approval of the Ethics Committee of Huaihe Hospital of Henan University.

### Cell culture and treatment

Human neuroblastoma cell line SH-SY5Y was purchased from America Type Culture Colletion (ATCC, Manassas, VA, USA). Cells were incubated in high-glucose DMEM medium (Thermo Fisher Scientific, Waltham, MA, USA) supplemented with 10% fetal bovine serum, 100 U/ml penicillin (Invitrogen, Carlsbad, CA, USA) and 100 μg/ml streptomycin (Invitrogen) at 37 °C in 5% CO_2_ incubator. According to the specific experimental requirements, SH-SY5Y cells were treated with 1 mM MPP^+^ (Sigma-Aldrich) for 48 h to establish the in vitro PD model.

### Cell transfection

The cDNA sequence of SNHG1 or PTEN was amplified and inserted into pcDNA3.1 vector (Invitrogen) to create SNHG1-overexpressing plasmid (pcDNA-SNHG1) or PTEN-overexpressing plasmid (pcDNA-PTEN), with pcDNA3.1 empty vector (pcDNA) as the negative control. siRNA antagonistic to SNHG1 (si-SNHG1), siRNA antagonistic to PTEN (si-PTEN), and scrambled negative control (si-NC) were obtained from GenePharma (Shanghai, China). miR-153-3p mimics (miR-153-3p), miR-153-3p antagonist (anti-miR-153-3p), and scrambled oligomer sequences (miR-NC, anti-miR-NC) were designed and synthesized by Sangon Biotech (Shanghai, China). SH-SY5Y cells (1 × 10^5^/ml) were inoculated on the 6-well plates and incubated to 80% confluence. Transfection with plasmids and/or oligonucleotides was carried out using Lipofectamine 2000 (Invitrogen), followed by treatment with 1 mM MPP^+^ for 48 h.

### qRT-PCR

Total RNA was extracted from the brain tissues and SH-SY5Y cells by using the Trizol Kit (Invitrogen) according to the manufacturer’s procedures. First strand of complementary DNA (cDNA) was synthesized by SuperScript First Strand cDNA System (Invitrogen). With GAPDH as the internal control, the SNHG1 expression was detected by using SYBR Green Real-Time PCR Master Mixes (Applied Biosystems, Foster City, CA, USA). With U6 small nuclear RNA (snRNA) as the reference, the expression level of miR-153-3p was quantitatively analyzed by using MirVana™ qRT-PCR miRNA Detection Kit (Ambion, Austin, TX, USA). qRT-PCR reactions were performed on the ABI 7500 RealTime PCR Systems (Applied Biosystems, Foster City). The primer sequences were listed as follows: SNHG1, 5′-GACAAGACCCAUCUUUAUGCAA-3′(forward)/5′-UUGCAUAAAGAUGGGUCUUGUC-3′(reverse); miR-153-3p, 5′-UUGCAUAGUCACAAAAGUGAUC-3′(forward)/5′-GAUCACUUUUGUGACUAUGCAA-3′(reverse); GAPDH, 5′-GTCAACGGATTTGGTCTGTATT-3′(forward)/5′-AGTCTTCTGGGTGGCAGTGAT-3′(reverse); U6, 5′-TGCGGGTGCTCGCTTCGCAGC-3′(forward)/5′-CCAGTGCAGGGTCCGAGGT-3′(reverse). Relative gene expression levels were calculated by the 2^−∆∆CT^ method.

### Cell viability assay/MTT assay

MTT assay was conducted to detect the viability of SH-SY5Y cells. Briefly, SH-SY5Y cells (1 × 10^5^) were planted on the 96-well plates. At indicated time points, 10 μl MTT stock solution (Sigma-Aldrich, 12 mM) was added into each well for another 4 h incubation at 37 °C. Then, the supernatant was removed and 150 μl dimethyl sulfoxide (DMSO) was added to dissolve the formazan crystals. The absorption value at 490 nm was detected by a microplate reader (Bio-Rad Laboratories, Hercules, CA, USA).

### Cell apoptosis assay

Annexin V-fluorescein isothiocyanate (FITC)/propidine iodide (PI) staining kit (BD Biosciences, San Diego, CA, USA) was used for determining cell apoptosis in accordance with the manufacturer’s instruction. Briefly, 0.05% trypsin (Sigma-Aldrich) was supplemented to digest cells at 37 °C followed by centrifugation (200×g for 3 min). Cells were washed twice with 0.01 M pre-cooling PBS and re-suspended with 1× binding buffer. Subsequently, cells were incubated with Annexin V-FITC and PI for 20 min at room temperature in the dark. Finally, apoptotic rate was measured using a FACScan flow cytometry (BD Biosciences) equipped with Cell Quest software (BD Biosciences).

### Western blot

Total proteins were extracted from SH-SY5Y cells using RIPA lysis buffer (Thermo Fisher Scientific). Equal amount (30 μg) of total proteins was mixed with 12% SDS-polyacrylamide gel (SDS-PAGE), separated by gel electrophoresis, and then transferred onto the PVDF membrane (Millipore, Billerica, MA, USA). Following incubation in the blocking buffer (0.1% Tween-20 Tris buffer solution containing 5% skim milk; TBST) at room temperature for 1 h, the membrane was cultivated overnight with moderate concentrations of primary antibodies against PTEN, AKT, p-AKT, mTOR, p-mTOR and β-actin (Cell Signaling technology, Boston, Massachusetts, USA) at 4 °C. Subsequently, the membrane was washed with TBST for three times and probed with appropriate amount of horseradish peroxidase (HRP)-conjugated secondary antibody for 1 h at room temperature. After being washed with TBST for three times, the protein band signals were detected using enhanced chemiluminescence luminator (Bio-Rad Laboratories) and quantified by Quantity One software version 4.4 (Bio-Rad Laboratories). β-actin was used as a reference protein.

### Luciferase activity assay

The wild-type or mutant SNHG1 fragments containing the miR-153-3p binding site were amplified by PCR and then inserted into the downstream region of the luciferase reporter vector pGL3 (Promega, Madison, WI, USA) to construct the recombinant luciferase reporter labeled as SNHG1-wt and SNHG1-mut, respectively. Similarly, 3′ non-translated regions of the wild-type or mutant PTEN containing the putative miR-153-3p binding site were synthesized and subcloned into the downstream region of pGL3 vector to obtain PTEN-wt and PTEN-mut, respectively. Then, the constructed luciferase reporter (SNHG1-wt, SNHG1-mut, PTEN-wt, or PTEN-mut) was co-transfected into SH-SY5Y cells together with miR-NC or miR-153-3p. At 48 h after transfection, the firefly luciferase activity was measured by Luciferase Reporter Assay System (Promega) and normalized to Renilla luciferase activity.

### RNA binding protein immunoprecipitation (RIP) assay

EZ-Magna RIP RNA Binding Protein Immunoprecipitation Kit (Millipore, Billerica, MA, USA) was utilized to analyze the binding specificity between SNHG1 and miR-153-3p. Briefly, SH-SY5Y cells were washed with PBS and lysed in the RIP lysis buffer containing protease and ribonuclease inhibitors. Subsequently, 100 μl of cell lysate was cultured with RIP buffer containing magnetic beads coupled with human anti-Argonaute 2 antibody (Ago2; Millipore) or standard mouse immunoglobulin G (IgG; Millipore). Then, the mixture was co-cultured with protease K to decompose the protein content and facilitate the isolation of immunoprecipitated RNA. qRT-PCR was performed to detect the levels of SNHG1 and miR-153-3p within the precipitates.

### RNA pull-down assay

SH-SY5Y cells were transfected with biotinylated SNHG1 (Bio-SNHG1). A biotinylated lncRNA that is not complimentary to miR-153-3p was taken as the negative control (Bio-NC). After 48 h, cell lysates were incubated with M-280 streptavidin magnetic beads (Invitrogen). qRT-PCR assay was applied to examine the level of miR-153-3p in the beads-bound RNA complexes.

### Statistical analysis

All data were reported as mean ± standard deviation (S.D.) of three independent experiments. SPSS19.0 software (SPSS Inc., Chicago, IL, USA) was used for statistical analysis. Statistical significance of the differences was evaluated by Student *t* test or one-way ANOVA. *P *< 0.05 was considered as a significant difference.

## Results

### Overexpression of SNHG1 inhibited viability and induced apoptosis in MPP^+^-treated SH-SY5Y cells

**Fig. 1:**
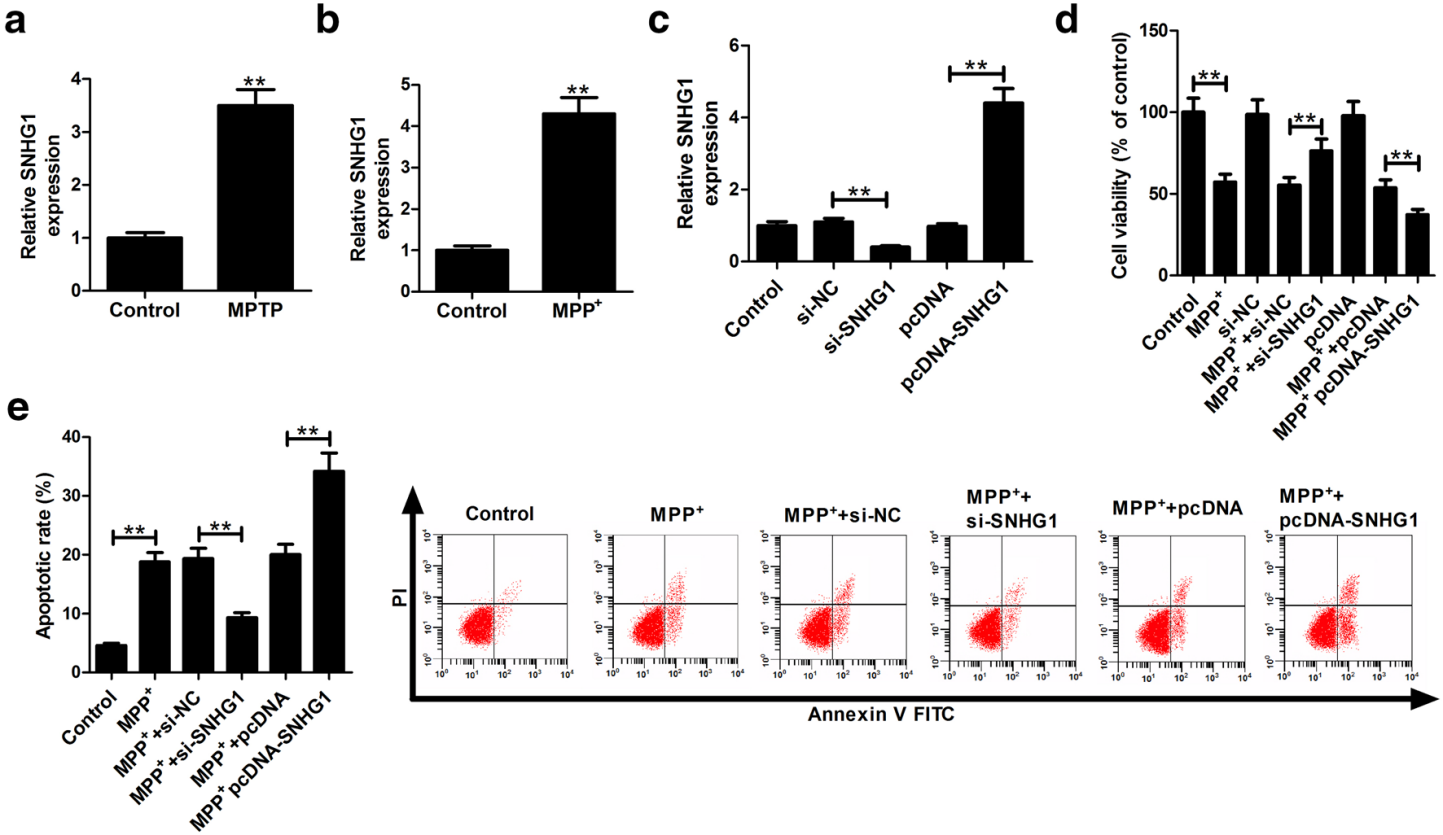
Overexpression of SNHG1 inhibits the viability and promotes the apoptosis in SH-SY5Y cells treated with MPP^+^. **a** Expression levels of SNHG1 in midbrain of MPTP-induced PD mice. **b** Expression levels of SNHG1 in SH-SY5Y cells after treatment with 1 mM MPP^+^ for 48 h. **c** Effects of si-SNHG1 or pcDNA-SNHG1 on the expression level of SNHG1 in SH-SY5Y cells were measured by qRT-PCR. **d** MTT assay was performed to detect the viability in MPP^+^-treated SH-SY5Y cells after transfection with si-SNHG1 or pcDNA-SNHG1. **e** Flow cytometry analysis was conducted to examine the effects of SNHG1 overexpression or knockdown on apoptosis in SH-SY5Y cells in the presence of MPP^+^. **P < 0.01

To investigate the function of SNHG1 in PD, MPTP and MPP^+^ were individually applied to induce the PD phenotype in vivo and in vitro. qRT-PCR results showed that SNHG1 expression was significantly increased in the midbrain of MPTP-induced PD mice compared with control mice (Fig. 1a). Also, SNHG1 expression was found to be up-regulated in SH-SY5Y cells treated with MPP^+^ when compared to control group (Fig. 1b). To illustrate the potential effects of SNHG1 on the PD pathogenesis, SH-SY5Y cells were transfected with si-SNHG1 or pcDNA-SNHG1 to down-regulate or up-regulate the SNHG1 expression (Fig. 1c). Then, non-transfected or transfected SH-SY5Y cells were treated with 1 mM MPP^+^ for 48 h. MTT assay manifested that SNHG1 knockdown increased the viability of MPP^+^-treated SH-SY5Y cells, while SNHG1 overexpression decreased the SH-SY5Y cell viability in the presence of MPP^+^ (Fig. 1d). Flow cytometry assay disclosed that MPP^+^-induced apoptosis was greatly reduced by silencing of SNHG1, while enforced expression of SNHG1 further aggravated MPP^+^-induced apoptosis in SH-SY5Y cells (Fig. 1e). Thus, high abundance of SNHG1 inhibited viability and enhanced apoptosis in MPP^+^-intoxicated SH-SY5Y cells.

### SNHG1 sponged miR-153-3p to suppress its expression

**Fig. 2:**
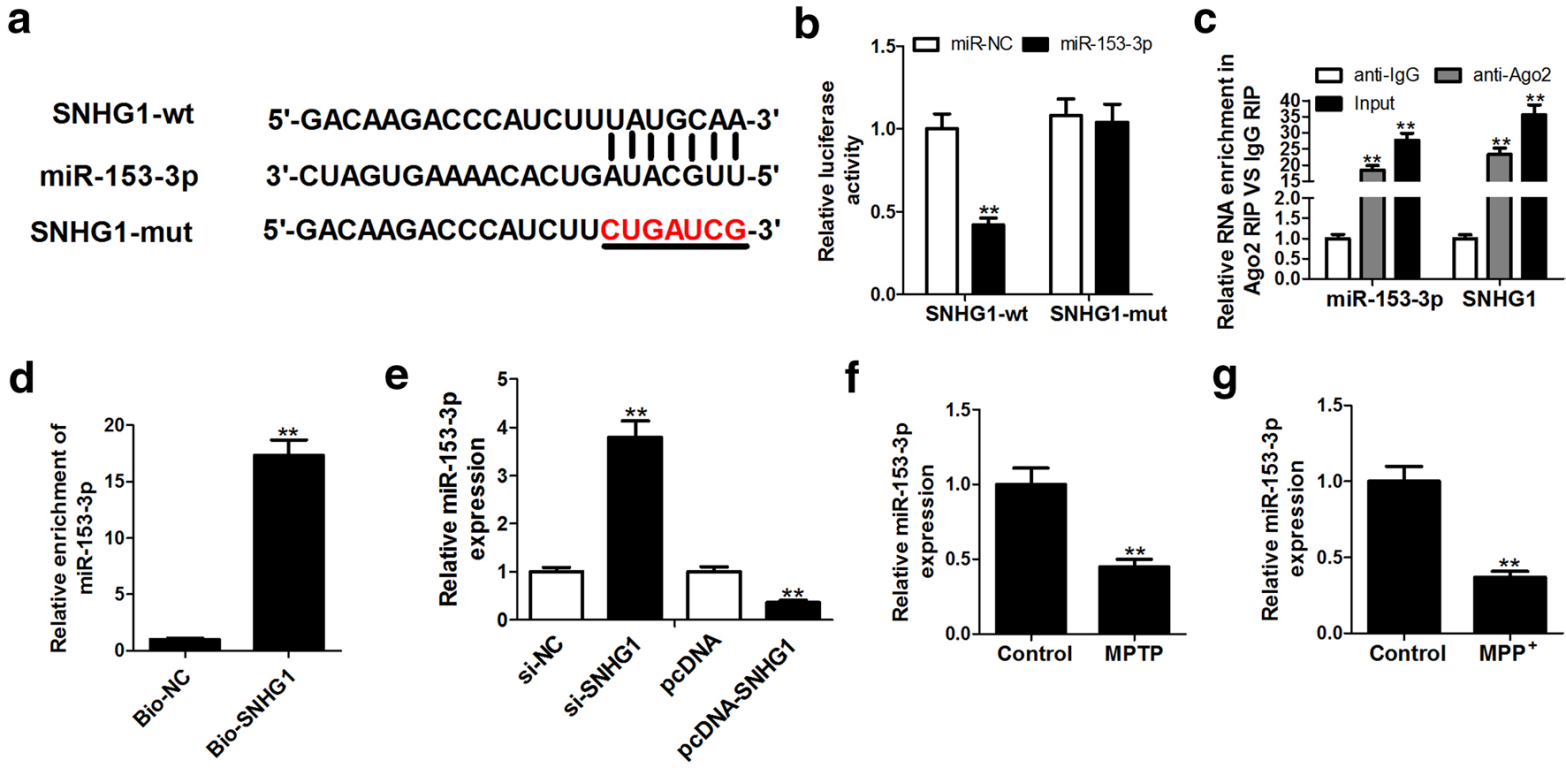
SNHG1 functions as the endogenous sponge of miR-153-3p and inhibits its expression in SH-SY5Y cells. **a** Wild-type or mutant SNHG1 transcript containing putative complementary recognition site of miR-153-3p. **b** Luciferase activity of SH-SY5Y cells co-transfected with SNHG1-wt or SNHG1-mut reporter and miR-153-3p or miR-NC was determined by luciferase reporter assay. **c** Correlation between SNHG1 and miR-153-3p in SH-SY5Y cell lysates was determined by RIP analysis with Ago2 antibody, IgG antibody was used as a negative control. **d** RNA pull-down analysis was conducted in SH-SY5Y cells using biotin-labeled SNHG1, followed by the determination of miR-153-3p expression by qRT-PCR. **e** Expression level of miR-153-3p in SH-SY5Y cells transfected with pcDNA-SNHG1, si-SNHG1 or the corresponding controls was detected by qRT-PCR analysis. Expression levels of miR-153-3p in midbrain of MPTP-induced PD mice **f** and MPP^+^-treated SH-SY5Y cells **g** were measured by qRT-PCR analysis. **P < 0.01

To elucidate the possible molecular mechanisms of SNHG1 involved in PD progression, the bioinformatics tool miRcode (http://www.mircode.org/) was used to predict the miRNAs containing the complementary binding sequences of SNHG1. As presented in Fig. 2a, miR-153-3p was found to be able to bind with SNHG1. To further validate the prediction and investigate the association between SNHG1 and miR-153-3p within SH-SY5Y cells, the wild-type or mutant SNHG1 fragments containing the miR-153-3p binding site were inserted into the pGL3 vector. Results of luciferase analysis exhibited that the miR-153-3p overexpression in SH-SY5Y cells repressed the luciferase activity of SNHG1-wt, but its inhibitory effects on the luciferase activity of SNHG1-mut were negligible (Fig. 2b). Besides, the potential binding between SNHG1 and miR-153-3p was further verified through the anti-Ago2 RIP analysis. In comparison with the anti-IgG control group, both SNHG1 and miR-153-3p levels were significantly enriched within the Ago2 precipitates (Fig. 2c). Moreover, results of RNA pull down analysis indicated that miR-153-3p was pulled down by biotin-labeled SNHG1 rather than by Bio-NC (Fig. 2d). To further identify the regulatory effects of SNHG1 on the expression of miR-153-3p, SH-SY5Y cells were transfected with si-SNHG1, pcDNA-SNHG1, or their corresponding controls. According to the qRT-PCR analysis, knockdown of SNHG1 significantly increased miR-153-3p expression, while up-regulation of SNHG1 remarkably suppressed miR-153-3p expression (Fig. 2e). In addition, based on the data from Fig. 2f, g, the expression levels of miR-153-3p were decreased in both in vivo and in vitro PD models. Hence, SNHG1 functioned as the endogenous sponge of miR-153-3p to inhibit its expression.

### miR-153-3p-mediated pro-proliferative and anti-apoptotic effects were abated following SNHG1 up-regulation in MPP^+^-induced SH-SY5Y cells

**Fig. 3:**
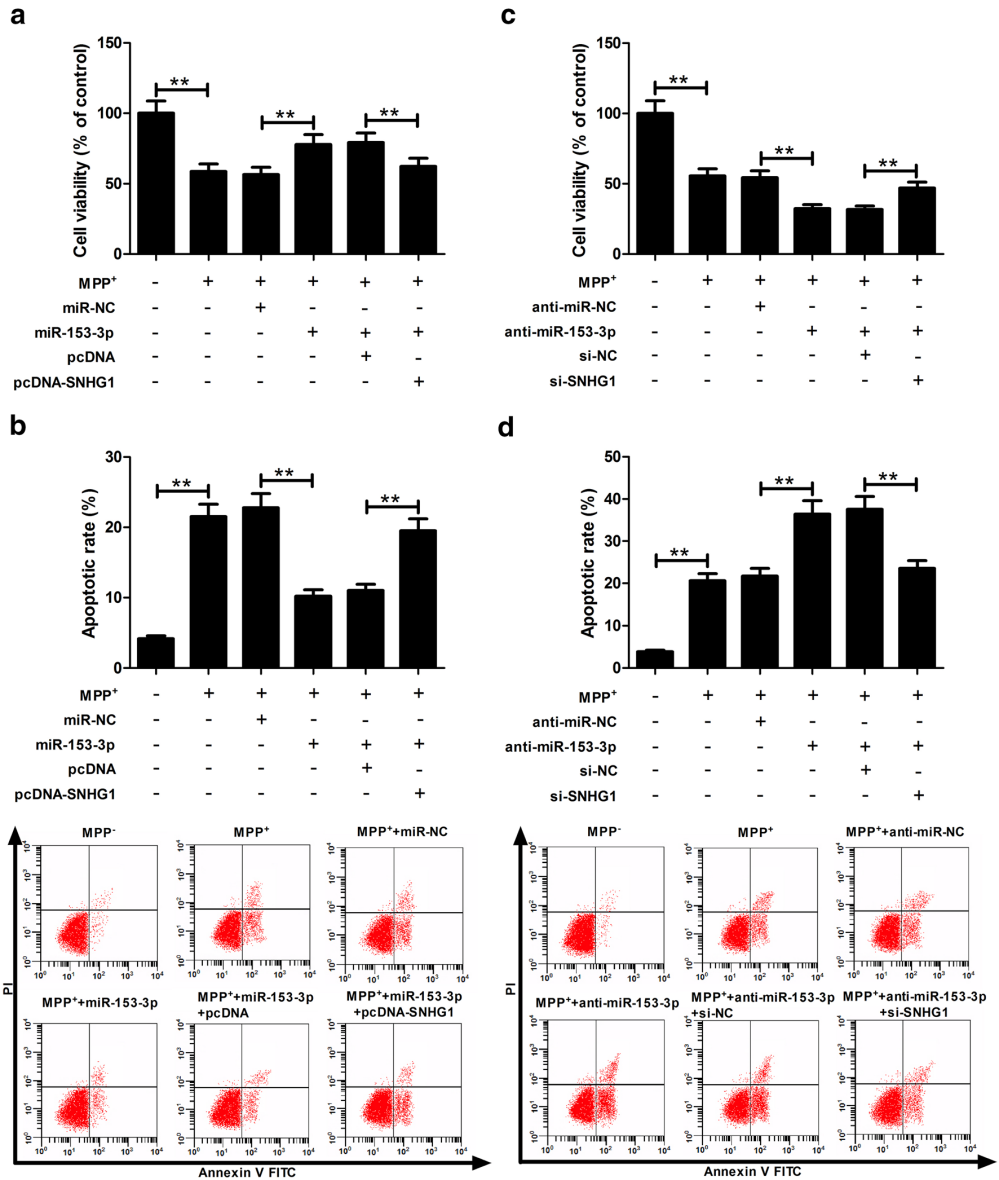
Increased expression of SNHG1 alleviates miR-153-3p-mediated pro-proliferative and anti-apoptotic effects in SH-SY5Y cell with MPP^+^ treatment. **a**, **b** Cell viability and apoptosis were detected in SH-SY5Y cells transfected with miR-NC, miR-153-3p, miR-153-3p + pcDNA, or miR-153-3p + pcDNA-SNHG1 after treatment with MPP^+^. **c**, **d** Cell viability and apoptosis were evaluated in SH-SY5Y cells transfected with anti-miR-NC, anti-miR-153-3p, anti-miR-153-3p + si-NC, or anti-miR-153-3p + si-SNHG1 after stimulation with MPP^+^. **P < 0.01

To further confirm whether the effects of SNHG1 on PD pathogenesis were mediated by miR-153-3p, SH-SY5Y cells were transfected with miR-NC, miR-153-3p, miR-153-3p + pcDNA, or miR-153-3p + pcDNA-SNHG1 prior to MPP^+^ treatment. Results indicated that miR-153-3p improved viability and decreased apoptosis in MPP^+^-induced SH-SY5Y cells, while these effects were remarkably reversed by SNHG1 up-regulation (Fig. 3a, b). Subsequently, SH-SY5Y cells were transfected with anti-miR-NC, anti-miR-153-3p, anti-miR-153-3p + si-NC, or anti-miR-153-3p + si-SNHG1 prior to MPP^+^ treatment. Results displayed that down-regulation of miR-153-3p lowered viability and facilitated apoptosis in MPP^+^-treated SH-SY5Y cells, however, these effects were evidently alleviated following SNHG1 knockdown (Fig. 3c, d). Together, miR-153-3p-mediated neuroprotective effects were impaired by SNHG1 overexpression in SH-SY5Y cells treated with MPP^+^.

### PTEN, regulated by SNHG1/miR-153-3p, inhibited viability and facilitated apoptosis in MPP^+^-treated SH-SY5Y cells

**Fig. 4:**
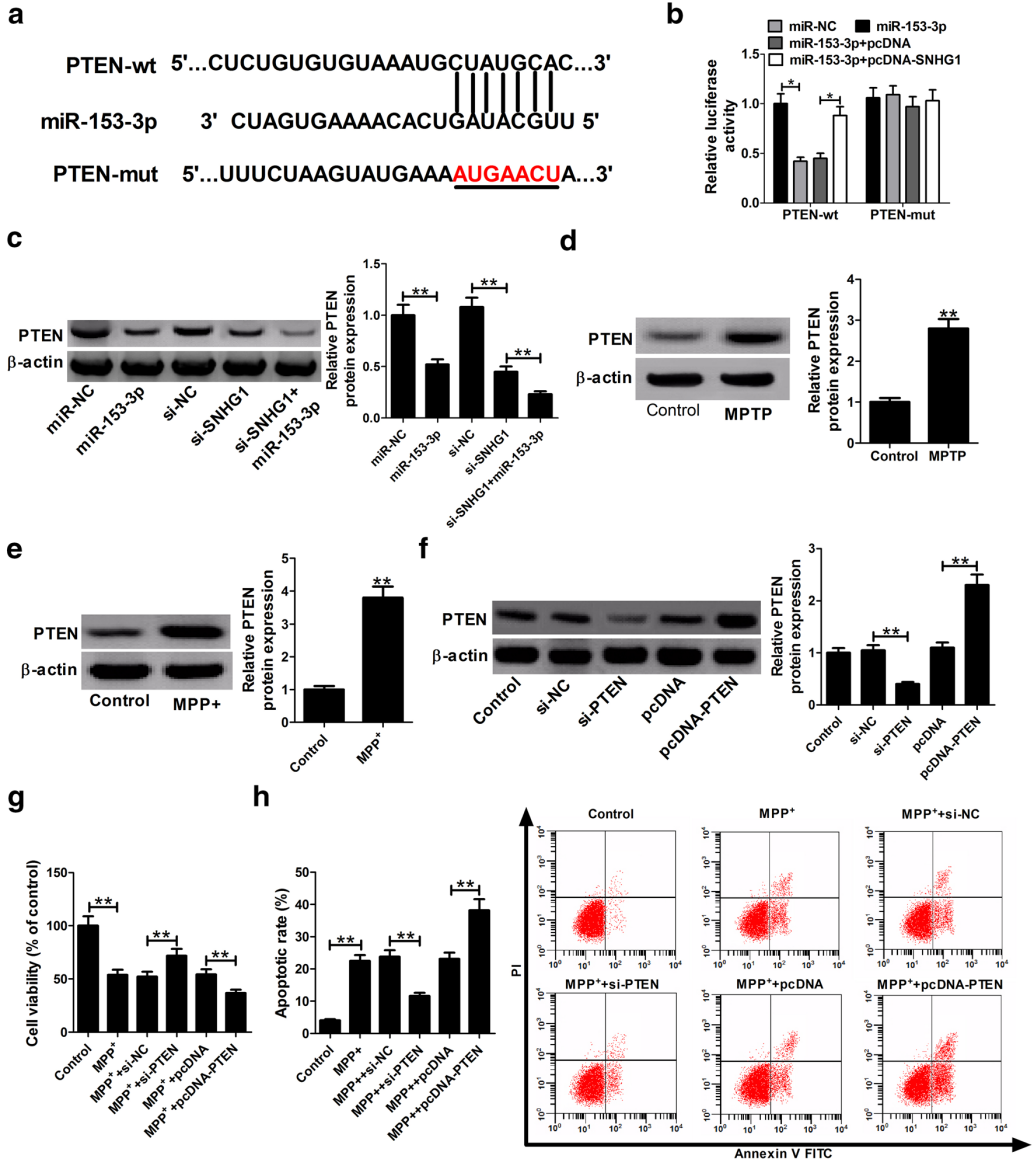
PTEN expression, regulated via the SNHG1/miR-153-3p signaling axis, inhibits viability and promotes apoptosis in SH-SY5Y cells treated with MPP^+^. **a** Presumed binding sites of miR-153-3p in the wild-type or mutant PTEN3′-UTR. **b** Luciferase activity of SH-SY5Y cells transfected with PTEN-wt or PTEN-mut reporter and miR-NC, miR-153-3p, miR153-3p + pcDNA or miR-153-3p + pcDNA-SNHG1 was determined by luciferase reporter assay. **c** Western blot assays were performed to assess the effects of miR-153-3p overexpression or SNHG1 knockdown on PTEN protein expression. Expression levels of PTEN protein in the midbrain of MPTP-induced PD mice **d** and MPP^+^-treated SH-SY5Y cells **e** were determined by western blotting. **f** Expression level of PTEN protein in SH-SY5Y cells after transfection with si-PTEN or pcDNA-PTEN. **g**, **h** Effect of PTEN overexpression or silencing on cell viability and apoptosis in the presence of MPP^+^. *P <0.05; **P < 0.01

Putative protein-coding gene targets of miR-153-3p were determined by employing the TargetScan (http://www.targetscan.org/vert_72/) and miRBase (http://www.mirbase.org/) database. Among the potential targets, PTEN was selected to further analysis for its involvement in the pathophysiology of the neurodegenerative disorders including PD [22]. To demonstrate the direct-binding between miR-153-3p and PTEN at the endogenous level, we constructed luciferase reporter vectors of PTEN-3′ UTR containing the wild-type or mutant binding sites of miR-153-3p (Fig. 4a) Then, PTEN-wt or PTEN-mut reporter was co-transfected with miR-NC, miR-153-3p, miR-153-3p + pcDNA, or miR-153-3p + pcDNA-SNHG1 into the SH-SY5Y cells. Results of luciferase reporter analysis revealed that miR-153-3p overexpression markedly suppressed the luciferase activity of PTEN-wt reporter, whereas this effect was attenuated by increasing the SNHG1 expression (Fig. 4b). However, little change was observed for the luciferase activity of PTEN-mut reporter following any transfection (Fig. 4b). Subsequently, the regulatory effect of miR-153-3p or SNHG1 on PTEN expression was detected in SH-SY5Y cells. Results presented that either miR-153-3p overexpression or SNHG1 knockdown significantly inhibited the protein expression of PTEN in SH-SY5Y cells, while the inhibitory effect was further enhanced after co-transfction with miR-153-3p and si-SNHG1 (Fig. 4c). Moreover, PTEN protein levels were found to be higher in both MPTP-induced PD mice (Fig. 4d) and MPP^+^-treated SH-SY5Y cells (Fig. 4e). These above results confirmed that SNHG1 promoted PTEN expression via serving as a sponge of miR-153-3p.

To elucidate the functions of PTEN in regulating the PD pathogenesis, si-PTEN or pcDNA-PTEN was introduced into SH-SY5Y cells to knockdown or enhance the PTEN expression before MPP^+^ treatment. As expected, PTEN protein expression was inhibited in SH-SY5Y cells transfected with si-PTEN, and was increased after transfection with pcDNA-PTEN (Fig. 4f). Functionally, knockdown of PTEN resulted in the improvement of cell viability and the reduction of apoptosis, while up-regulation of PTEN exerted the opposite effects in the presence of MPP^+^ (Fig. 4g, h). In short, PTEN, a downstream target of SNHG1/miR-153-3p, repressed viability and facilitated apoptosis in MPP^+^-intoxicated SH-SY5Y cells.

### SNHG1 regulated PTEN/AKT/mTOR signaling pathway in SH-SY5Y cells by targeting miR-153-3p

**Fig. 5:**
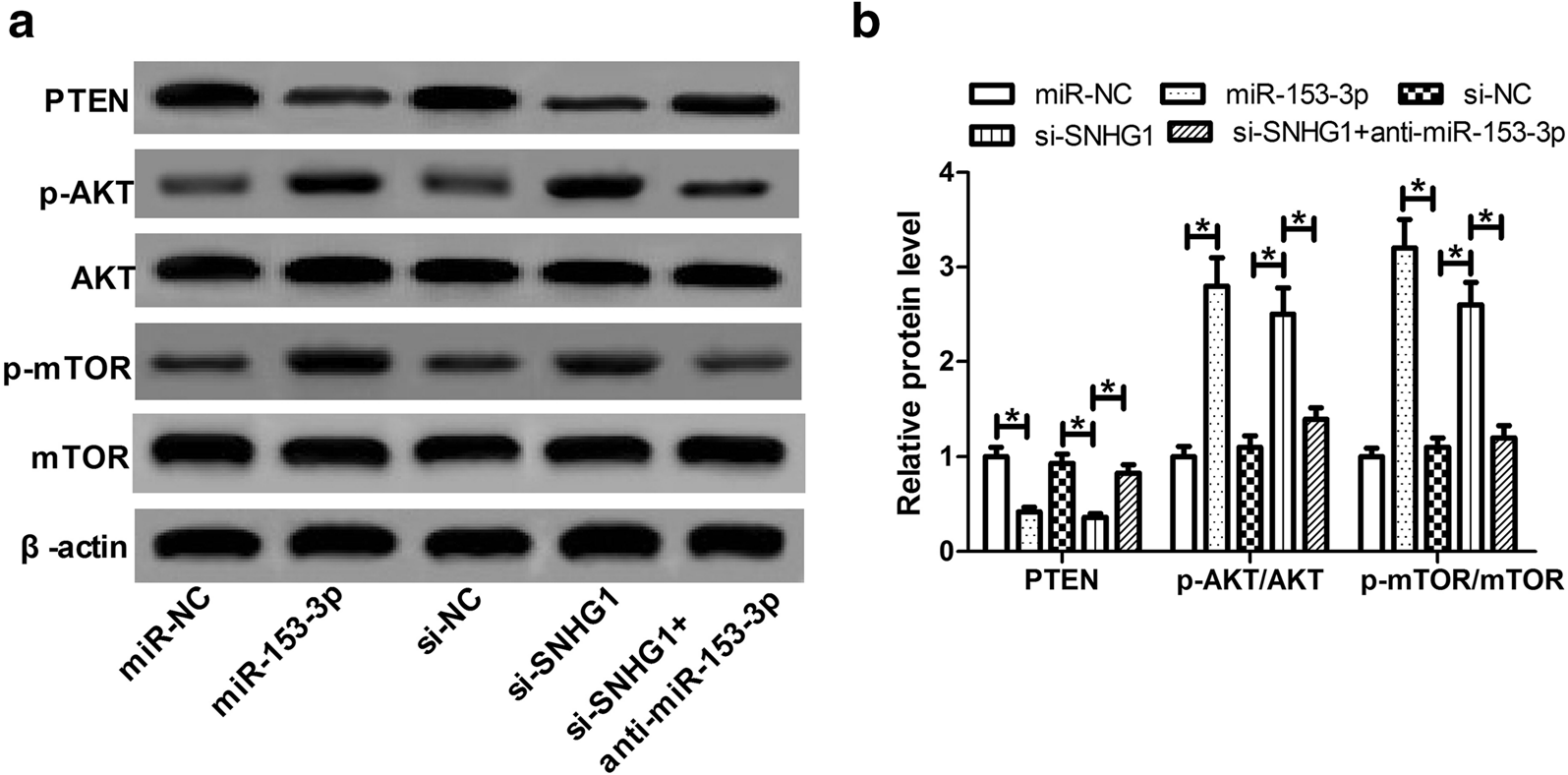
SNHG1 regulated PTEN/AKT/mTOR signaling pathway in SH-SY5Y cells by targeting miR-153-3p. **a** Western blot analysis was conducted to evaluate the protein expressions of PTEN, AKT, p-AKT, mTOR and p-mTOR in SH-SY5Y cells transfected with miR-153-3p, si-SNHG1 or si-SNHG1 + anti-miR-153-3p. **b** Quantification analysis of the protein levels of PTEN, p-AKT/AKT, and p-mTOR/mTOR. *P <0.05

PTEN/Akt/mTOR signaling pathway was previously demonstrated to be implicated in PD progression [23]. Thus, we further investigated whether SNHG1 could affect PTEN/AKT/mTOR signaling in SH-SY5Y cells through targeting miR-153-3p. Western blot assay showed that miR-153-3p overexpression or SNHG1 knockdown significantly lowered the protein level of PTEN while increased the protein levels of p-AKT and p-mTOR (Fig. 5a, b). Moreover, SNHG1 knockdown-mediated change of protein levels (PTEN, p-AKT and p-mTOR) was evidently reversed by suppression of miR-153-3p. Collectively, these data suggested that SNHG1 regulated PTEN/AKT/mTOR signaling pathway in SH-SY5Y cells via targeting miR-153-3p.

## Discussion

PD, the second most prevalent neurodegenerative disorder, is characterized by progressive bradykinesia, tremor, rigidity, and postural instability [24]. In the U.S., the PD incidence is expected to steadily increase, reaching to about 2 million until 2030 [25]. Loss and death of dopaminergic neurons in SNpc has been considered as the primary cause of the cardinal motor symptoms of PD [26]. With a high lipid solubility and a great accessibility into the brain, MPTP can be catalyzed by monoamine oxidase B located in the outer membrane of glial cells to produce MPP^+^ [27]. MPP^+^, the specific inhibitor of mitochondrial oxidative respiratory chain complex I, reduces ATP generation and induces DA neuron apoptosis [28]. Thus, MPTP and MPP + have been widely accepted as the typical neurotoxin to establish PD models in vivo and in vitro in a large number of researches.

In recent years, more and more researchers are focusing on the association of lncRNA in the pathological and behavioral changes of neurodegenerative diseases, including PD [29]. For instance, lncRNA-p21 regulated MPP^+^-induced neuronal injury by promoting TRPM2 expression through acting as a miR-625 sponge in SH-SY5Y cells [30]. LncRNA NEAT1 exacerbated MPTP-induced autophagy in PD via stabilizing PINK1 protein [31]. SNHG1 was previously found to facilitate the PD progression by affecting α-synuclein aggregation and neuroinflammation via different mechanisms [19, 20]. This study aims to explore the biological function and specific mechanism of SNHG1 in regulating neuron apoptosis in PD.

In this study, we found that SNHG1 expression was up-regulated in both MPTP-induced PD mice and MPP^+^-treated SH-SY5Y cells, which was in accordance with a previous report [19]. SNHG1 overexpression suppressed viability and enhanced apoptosis in MPP^+^-treated SH-SY5Y cells, while knockdown of SNHG1 exhibited the opposite effects. Similarly, a recent investigation reported that SNHG1 enhanced MPP^+^-induced cytotoxicity and reactive oxygen species (ROS) production by modulation of miR-15b-5p/GSK3β axis [32].

The ceRNA hypothesis has been widely recognized as a novel regulatory mechanism pertinent to lncRNAs [33]. Thus, we further clarified the possible regulatory pathways of SNHG1 in PD. Based on the data from the online bioinformatic databases, miR-153-3p was predicted to possess the complementary binding sites within SNHG1. As one of the brain-specific miRNAs, miR-153-3p was previously reported to play critical roles in neuronal patterning and neurotransmission [34]. miR-153 expression was found to be decreased in AD brain specimens, and dysregulation of miR-153 was associated with AD etiology [35, 36]. Moreover, miR-153 was previously found to protect neurons from cell death by interfering with the MPP^+^-induced blockage of mTOR signaling [37]. Also, miR-153 resulted in a significant reduction of endogenous α-synuclein, one of the major contributors in PD pathogenesis [38, 39]. Here, luciferase reporter, RIP and RNA pull-down experiments demonstrated that SNHG1 could directly bind with miR-153-3p. In addition, miR-153-3p expression was increased by SNHG1 knockdown, while was reduced following SNHG1 up-regulation in SH-SY5Y cells. Furthermore, we found that miR-153-3p expression was down-regulated in both in vivo and in vitro PD models. Above all, we draw the conclusion that SNHG1 served as an endogenous sponge to inhibit the expression of miR-153-3p.

Having identified the direct association between SNHG1 and miR-153-3p, we presumed that SNHG1 may contribute to PD progression through its negative regulatory effects on miR-153-3p. Function assays revealed that enforced expression of miR-153-3p improved viability and suppressed apoptosis in MPP^+^-treated SH-SY5Y cells, on the contrary, down-regulation of miR-153-3p lowered viability and facilitated apoptosis. What’s more, overexpression of SNHG1 mitigated the pro-proliferative and anti-apoptotic effects meditated by miR-153-3p. That is to say, SNHG1 could directly act on miR-153-3p and perform as the pathogenic factor during the PD progression by negatively regulating the expression level of miR-153-3p. Definitely, the function mechanism of SNHG1 would be identified in PD patients to illustrate the clinical effect and significance of aberrantly-expressed SNHG1, thus further elucidating the PD pathogenesis and providing guidance for the application of target-directed therapy.

Subsequent TargetScan prediction and luciferase reporter experiments verified that PTEN was a direct target of miR-153-3p. PTEN, a tumor suppressor, regulates cell growth and apoptosis, energy metabolism, signal transduction, cellular architecture and motility in both cancers and neurodegenerative diseases [40, 41]. PTEN could act as a vital mediator of ROS production and neuronal death, highlighting its possibility as a potential therapeutic target for neurodegenerative diseases [42, 43]. In our work, the expression level of PTEN protein in brain tissue cells of MPTP-induced PD mice and SH-SY5SY cells treated by MPP^+^ were both significantly increased. More importantly, we found that SNHG1 functioned as the ceRNA to sponge miR-153-3p in SH-SY5Y cells to positively regulate the PTEN expression. By analyzing the functions in depth, it’s evident that enforced expression of PTEN remarkably inhibited the viability and enhanced apoptosis in MPP^+^-induced SH-SY5Y cells. Consistently, a recent document elucidated that decreasing nuclear accumulation of PTEN may halt the neurodegeneration in PD [44]. Furhtermore, SNHG1 activated PTEN/AKT/mTOR signaling pathway in SH-SY5Y cells by targeting miR-153-3p. Consistently, PTEN was previously found to participate in PD process via affecting Akt/mTOR signaling pathway [45, 46].

## Conclusion

It was demonstrated that SNHG1 expression was up-regulated in both MPTP-induced PD mice and MPP^+^-treated SH-SY5Y cells. Mechanism analysis showed that knockdown of SNHG1 could inhibit MPP^+^-induced cytotoxicity in SH-SY5Y cells via the miR-153-3p/PTEN pathway. This study is conducive to understanding the pathogenesis and directing the clinical treatment in PD. Considering that there might be multiple targets or pathways for SNHG1, it’s essential to deeply explore the other possible functional mechanisms and interactions.

## Data Availability

Not applicable.

## Abbreviations

PD: Parkinson’s disease
MPTP: 1-methyl-4-phenyl-1,2,3,6-tetrahydropyridine-hydrochloride
PTEN: phosphate and tension homology deleted on chromosome ten
AD: Alzheimer’s disease
lncRNAs: long non-coding RNAs
CNS: central nervous system
MPP^+^: 1-methyl-4-phenylpyridinium
SNHG1: small molecule RNA host gene 1
FITC: fluorescein isothiocyanate

## References

[1] Schapira AH (2008). Mitochondria in the aetiology and pathogenesis of Parkinson’s disease. Lancet Neurol.

[2] Kalia LV, Lang AE (2015). Parkinson’s disease. Lancet.

[3] Rodriguez-Oroz MC, Jahanshahi M, Krack P, Litvan I, Macias R, Bezard E (2009). Initial clinical manifestations of Parkinson’s disease: features and pathophysiological mechanisms. Lancet Neurol.

[4] Fleming SM (2017). Mechanisms of gene-environment interactions in Parkinson’s disease. Curr Environ Health Rep.

[5] Poewe W, Seppi K, Tanner CM, Halliday GM, Brundin P, Volkmann J (2017). Parkinson disease. Nat Rev Dis Primers.

[6] Maass PG, Luft FC, Bahring S (2014). Long non-coding RNA in health and disease. J Mol Med (Berl).

[7] Maniati MS, Maniati M, Yousefi T, Ahmadi-Ahangar A, Tehrani SS (2019). New insights into the role of microRNAs and long noncoding RNAs in most common neurodegenerative diseases. J Cell Biochem.

[8] Wan P, Su W, Zhuo Y (2017). The role of long noncoding RNAs in neurodegenerative diseases. Mol Neurobiol.

[9] Xu X, Zhuang C, Wu Z, Qiu H, Feng H, Wu J (2018). LincRNA-p21 inhibits cell viability and promotes cell apoptosis in Parkinson’s disease through activating alpha-synuclein expression. Biomed Res Int.

[10] Lin Q, Hou S, Dai Y, Jiang N, Lin Y (2019). LncRNA HOTAIR targets miR-126-5p to promote the progression of Parkinson’s disease through RAB3IP. Biol Chem.

[11] Huang L, Jiang X, Wang Z, Zhong X, Tai S, Cui Y (2018). Small nucleolar RNA host gene 1: a new biomarker and therapeutic target for cancers. Pathol Res Pract.

[12] Thin KZ, Tu JC, Raveendran S (2019). Long non-coding SNHG1 in cancer. Clin Chim Acta.

[13] Wang Q, Li Q, Zhou P, Deng D, Xue L, Shao N (2017). Upregulation of the long non-coding RNA SNHG1 predicts poor prognosis, promotes cell proliferation and invasion, and reduces apoptosis in glioma. Biomed Pharmacother.

[14] Liu L, Shi Y, Shi J, Wang H, Sheng Y, Jiang Q (2019). The long non-coding RNA SNHG1 promotes glioma progression by competitively binding to miR-194 to regulate PHLDA1 expression. Cell Death Dis.

[15] Wang Z, Wang R, Wang K, Liu X (2018). Upregulated long noncoding RNA SNHG1 promotes the angiogenesis of brain microvascular endothelial cells after oxygen–glucose deprivation treatment by targeting miR-199a. Can J Physiol Pharmacol.

[16] Yang X, Zi XH (2019). LncRNA SNHG1 alleviates OGD induced injury in BMEC via miR-338/HIF-1α axis. Brain Res.

[17] Zhang L, Luo X, Chen F, Yuan W, Xiao X, Zhang X (2018). LncRNA SNHG1 regulates cerebrovascular pathologies as a competing endogenous RNA through HIF-1alpha/VEGF signaling in ischemic stroke. J Cell Biochem.

[18] Kraus TFJ, Haider M, Spanner J, Steinmaurer M, Dietinger V, Kretzschmar HA (2017). Altered long noncoding RNA expression precedes the course of Parkinson’s disease—a preliminary report. Mol Neurobiol.

[19] Chen Y, Lian YJ, Ma YQ, Wu CJ, Zheng YK, Xie NC (2018). LncRNA SNHG1 promotes alpha-synuclein aggregation and toxicity by targeting miR-15b-5p to activate SIAH1 in human neuroblastoma SH-SY5Y cells. Neurotoxicology.

[20] Cao B, Wang T, Qu Q, Kang T, Yang Q (2018). Long noncoding RNA SNHG1 promotes neuroinflammation in Parkinson’s disease via regulating miR-7/NLRP3 pathway. Neuroscience.

[21] Tay Y, Rinn J, Pandolfi P (2014). The multilayered complexity of ceRNA crosstalk and competition. Nature..

[22] Ogino M, Ichimura M, Nakano N, Minami A, Kitagishi Y, Matsuda S (2016). Roles of PTEN with DNA repair in Parkinson’s Disease. Int J Mol Sci.

[23] Li W, Jiang Y, Wang Y, Yang S, Bi X, Pan X (2018). MiR-181b regulates autophagy in a model of Parkinson’s disease by targeting the PTEN/Akt/mTOR signaling pathway. Neurosci Lett.

[24] Borrione P, Tranchita E, Sansone P, Parisi A (2014). Effects of physical activity in Parkinson’s disease: a new tool for rehabilitation. World J Methodol.

[25] Dorsey ER, Constantinescu R, Thompson JP, Biglan KM, Holloway RG, Kieburtz K (2007). Projected number of people with Parkinson disease in the most populous nations, 2005 through 2030. Neurology.

[26] Surmeier DJ (2018). Determinants of dopaminergic neuron loss in Parkinson’s disease. FEBS J.

[27] Singer TP, Salach JI, Castagnoli N, Trevor A (1986). Interactions of the neurotoxic amine 1-methyl-4-phenyl-1,2,3,6-tetrahydropyridine with monoamine oxidases. Biochem J.

[28] Fiskum G, Starkov A, Polster BM, Chinopoulos C (2003). Mitochondrial mechanisms of neural cell death and neuroprotective interventions in Parkinson’s disease. Ann N Y Acad Sci.

[29] Wang DQ, Fu P, Yao C, Zhu LS, Hou TY, Chen JG (2018). Long non-coding RNAs, novel culprits, or bodyguards in neurodegenerative diseases. Mol Ther Nucleic Acids.

[30] Ding XM, Zhao LJ, Qiao HY, Wu SL, Wang XH (2019). Long non-coding RNA-p21 regulates MPP^+^-induced neuronal injury by targeting miR-625 and derepressing TRPM2 in SH-SY5Y cells. Chem Biol Interact.

[31] Yan W, Chen ZY, Chen JQ, Chen HM (2018). LncRNA NEAT1 promotes autophagy in MPTP-induced Parkinson’s disease through stabilizing PINK1 protein. Biochem Biophys Res Commun.

[32] Xie N, Qi J, Li S, Deng J, Chen Y, Lian Y (2019). Upregulated lncRNA small nucleolar RNA host gene 1 promotes 1-methyl-4-phenylpyridinium ion-induced cytotoxicity and reactive oxygen species production through miR-15b-5p/GSK3beta axis in human dopaminergic SH-SY5Y cells. J Cell Biochem.

[33] Denzler R, Agarwal V, Stefano J, Bartel DP, Stoffel M (2014). Assessing the ceRNA hypothesis with quantitative measurements of miRNA and target abundance. Mol Cell.

[34] Wei C, Thatcher EJ, Olena AF, Cha DJ, Perdigoto AL, Marshall AF (2013). miR-153 regulates SNAP-25, synaptic transmission, and neuronal development. PLoS ONE.

[35] Long JM, Ray B, Lahiri DK (2012). MicroRNA-153 physiologically inhibits expression of amyloid-beta precursor protein in cultured human fetal brain cells and is dysregulated in a subset of Alzheimer disease patients. J Biol Chem.

[36] Liang C, Zhu H, Xu Y, Huang L, Ma C, Deng W (2012). MicroRNA-153 negatively regulates the expression of amyloid precursor protein and amyloid precursor-like protein 2. Brain Res.

[37] Fragkouli A, Doxakis E (2014). miR-7 and miR-153 protect neurons against MPP^+^-induced cell death via upregulation of mTOR pathway. Front Cell Neurosci.

[38] Doxakis E (2010). Post-transcriptional regulation of alpha-synuclein expression by mir-7 and mir-153. J Biol Chem.

[39] Je G, Kim YS (2017). Mitochondrial ROS-mediated post-transcriptional regulation of alpha-synuclein through miR-7 and miR-153. Neurosci Lett.

[40] Worby CA, Dixon JE (2014). PTEN. Annu Rev Biochem.

[41] Kim RH, Mak TW (2006). Tumours and tremors: how PTEN regulation underlies both. Br J Cancer.

[42] Zhu Y, Hoell P, Ahlemeyer B, Sure U, Bertalanffy H, Krieglstein J (2007). Implication of PTEN in production of reactive oxygen species and neuronal death in in vitro models of stroke and Parkinson’s disease. Neurochem Int.

[43] Diaz-Ruiz O, Zapata A, Shan L, Zhang Y, Tomac AC, Malik N (2009). Selective deletion of PTEN in dopamine neurons leads to trophic effects and adaptation of striatal medium spiny projecting neurons. PLoS ONE.

[44] Sekar S, Taghibiglou C (2018). Elevated nuclear phosphatase and tensin homolog (PTEN) and altered insulin signaling in substantia nigral region of patients with Parkinson’s disease. Neurosci Lett.

[45] Wang X, Pang L, Zhang Y, Xu J, Ding D, Yang T (2018). Lycium barbarum polysaccharide promotes nigrostriatal dopamine function by modulating PTEN/AKT/mTOR pathway in a methyl-4-phenyl-1,2,3,6-tetrahydropyridine (MPTP) murine model of Parkinson’s Disease. Neurochem Res.

[46] Ge H, Yan Z, Zhu H, Zhao H (2019). MiR-410 exerts neuroprotective effects in a cellular model of Parkinson’s disease induced by 6-hydroxydopamine via inhibiting the PTEN/AKT/mTOR signaling pathway. Exp Mol Pathol.

